# Rapid tests as a practical alternative to slide agglutination for the confirmation of *V. cholerae* O1

**DOI:** 10.1128/jcm.00433-25

**Published:** 2025-07-17

**Authors:** Piyash Bhattacharjee, Sonia T. Hegde, Ashraful Islam Khan, Imrul Kayes Nabil, Md. Naiem Hossain, Tahira Ahmed Rashmi, Mokibul Hassan Afrad, Md. Taufiqul Islam, Mohammad Ashraful Amin, Zahid Hasan Khan, Taufiqur Rahman Bhuiyan, Andrew S. Azman, Firdausi Qadri

**Affiliations:** 1 Infectious Diseases Division, International Centre for Diarrhoeal Disease Research, Bangladesh (icddr,b) 56291, Dhaka, Bangladesh; 2 Department of Epidemiology, Johns Hopkins Bloomberg School of Public Health 25802 , Baltimore, Maryland, USA; 3 Geneva Centre for Emerging Viral Diseases, Geneva University Hospitals 27230, Geneva, Switzerland; 4 Division of Tropical and Humanitarian Medicine, Geneva University Hospitals 27230, Geneva, Switzerland; Maine Medical Center Department of Medicine, Portland, Maine, USA

**Keywords:** rapid test, culture, cholera

## Abstract

Slide agglutination for serogroup and/or serotype identification is a crucial step for confirming cholera by culture. Rapid diagnostic tests (RDTs) are typically used directly on stool but are not considered sufficient for cholera confirmation. However, they may provide a practical alternative to the slide agglutination step of culture*,* as they are easy to use, store, and require minimal training. This study evaluates the concordance of *Vibrio cholerae* O1/O139 detection from presumptive colonies by slide agglutination with RDTs. Patients (≥1 year) with acute watery diarrhea at the icddr,b Dhaka hospital were enrolled. Stool samples were cultured on thiosulfate-citrate-bile salts-sucrose medium, and presumptive colonies were subcultured on Gelatin Agar medium. Isolates were then tested by slide agglutination and four commercial cholera RDTs. From 4 February 2024 through 24 May 2025, 1,638 patients with acute watery diarrhea were enrolled, 1,140 (70%) had presumptive *V. cholerae* colonies, and 482 (29%) were culture-confirmed as *V. cholerae* O1. We tested suspected *V. cholerae* colonies using Cholkit (n=1,140), SD Bioline (n=693), Crystal VC O1/O139 (n=693), and Crystal VC O1 (n=655) RDT kits. RDTs showed near-perfect concordance with slide agglutination. Using slide agglutination as the reference, the sensitivity of the RDT kits ranged from 99.3% to 99.6% and the specificity from 99.3% to 99.7%. RDTs offer a practical and potentially easier alternative to slide agglutination of presumptive *V. cholerae* colonies within typical cholera culture protocols. This may help to provide a pathway to quick confirmation of outbreaks in settings where lab facilities and reagents may be limited.

**IMPORTANCE:**

This study demonstrated that rapid diagnostic tests (RDTs) can effectively replace traditional slide agglutination methods to confirm suspected *Vibrio cholerae* colonies belonging to the O1 serogroup, which are responsible for the current seventh cholera pandemic. Comparing four RDTs to slide agglutination, we found almost perfect agreement, with sensitivity and specificity exceeding 99%. Since RDTs are cheaper, easier to use and store, and require less technical training, adopting them could significantly speed up cholera outbreak confirmation, especially in areas with limited laboratory resources. This practical shift could lead to faster responses to cholera outbreaks, ultimately improving public health surveillance and disease control globally.

## INTRODUCTION

Cholera continues as a global public health concern, causing significant mortality and morbidity in addition to economic strains in communities lacking safe water and sanitation. While not considered useful for individual-level clinical decision-making, laboratory confirmation of suspected cholera cases is critical for declaring outbreaks, tracking trends in incidence, and monitoring antimicrobial resistance patterns of the bacteria (1). Both polymerase chain reaction (PCR) and culture are considered gold-standard methods to confirm *Vibrio cholerae* O1/O139, though PCR is not widely used in settings where cholera transmission occurs (1, 2).

Stool culture-based diagnosis of *V. cholerae* involves first growing the bacteria in a selective medium and then confirming the serogroup and/or serotype of suspected bacterial colonies by conducting slide agglutination with antisera (2–4). The antisera needed to confirm cholera is expensive, has a short shelf life—especially compared to the recurrent frequency of cholera in some settings—and requires skilled personnel. Lack of antisera has been reported as a reason for delayed confirmation of outbreaks in several settings over the past decade. Due to the often-weak visual signal, the interpretation of agglutination tests can vary from person to person. Test interpretation can be influenced by uncorrected near-vision impairments, which is common in low-and-middle-income settings where cholera usually occurs (5).

Lateral flow immunoassay rapid diagnostic tests (RDTs) for cholera*,* which are based on the detection of the O-antigen of the bacterial lipopolysaccharide, are used and recommended by the WHO Global Task Force for Cholera Control (GTFCC) for surveillance of cholera directly from stool or rectal swabs (1). Though RDTs have been primarily developed to test for *V. cholerae* O1 (6, 7), a few tests simultaneously detect *V. cholerae* O1 and O139, the rare serogroup that can also cause outbreaks. Aside from use in standard surveillance systems, these tests may also offer an alternative, efficient, and perhaps more robust method to complete the final bacterial culture step of identifying serogroup/serotype from suspected bacterial colonies. Here, we compare this alternative use of RDTs with slide agglutination to confirm suspected colonies as *V. cholerae* O1/O139 as part of culture confirmation.

## MATERIALS AND METHODS

As part of a larger evaluation of RDT performance, we recruited suspected cholera cases (patients ≥1 year of age experiencing acute watery diarrhea with three or more non-bloody, loose stools and symptoms in the preceding 24 hours prior to the hospital visit) from the icddr,b diarrhea hospital in Dhaka, Bangladesh, from 4 February 2024 through 24 May 2025. After providing informed written consent/assent, study staff administered a short questionnaire and collected a stool sample from each case.

Within 1 hour of collection, stool samples from each case were cultured overnight on thiosulfate-citrate-bile salts-sucrose culture medium with one suspected colony then subcultured on gelatin agar culture medium. One suspected colony from the gelatin agar culture plate was then tested by slide agglutination with 10 µL of *V. cholerae* O1 Ogawa, Inaba, and O139 antisera. We put two to three presumptive colonies from the gelatin agar culture plate into the diluents provided with each of four lateral flow RDTs: Cholkit (Incepta, Bangladesh), SD Bioline (Abbott, Republic of Korea), Crystal VC O1/O139 (Arkay, India), and Crystal VC O1 (Arkay, India). Cholkit and Crystal VC O1 kits detect *V. cholerae* O1, and SD Bioline and Crystal VC O1/O139 detect both *V. cholerae* O1 and O139 (separately). All these tests are based on the detection of all or part of the O1/O139-specific lipopolysaccharide (LPS).

Additionally, PCR tests for *V. cholerae* O1 and O139 were performed (rfb O1, rfb O139, and ctxA primers) for further molecular-level confirmation on stored stool specimens (not on colonies), approximately 1 week after sample collection (8). The PCR results were used to further contextualize discrepant slide agglutination and RDT results. This study was started only using CholKit RDTs to compare to agglutination, but after promising preliminary results, we expanded this to all kits; thus, the numbers of tests performed for each test differed.

Data needed to reproduce analyses in this manuscript can be found at https://github.com/HopkinsIDD/rdt-agglutination.

## RESULTS

We recruited 1,638 suspected cholera cases from icddr,b hospital in Dhaka from 4 February 2024 through 24 May 2025, including 35% severely dehydrated (based on the Dhaka Score [9]), 37% under 5 years old, and 47% female. From these cases, 1,140 (70%) had stool with suspected *V. cholerae* colonies, and 482 (29%) were positive for *V. cholerae* O1 by culture, including slide agglutination. No samples were positive for *V. cholerae O139 by culture*.

We tested suspected *V. cholerae* colonies using Cholkit (n=1,140), SD Bioline (n=693), Crystal VC O1/O139 (n=693), and Crystal VC O1 (n=655) RDT kits. We found nearly perfect concordance of the slide agglutination results with all four RDTs, with only seven samples having discordant results (Table 1). Considering slide agglutination as the reference standard for confirmation of *V. cholerae* O1 among suspected colonies, the sensitivity across tests ranged from 99.3%–99.6%, and the specificity ranged from 99.3% to 99.7% (Table 2). Though our primary focus was on the detection of *V. cholerae* O1 as compared to slide agglutination, the specificity of RDTs for the detection of *V. cholerae* O139 within suspected culture colonies was 100% for SD Bioline and 99.6% for Crystal VC O1/O139.

**TABLE 1:** Diagnostics based on slide agglutination and RDT test results from suspected *V. cholerae* isolates in terms of detection of *Vibrio cholerae* O1 and O139^*a*^

		Cholkit		SD Bioline				Crystal VC O1/O139				Crystal VC O1	
		O1		O1		O139		O1		O139		O1	
		+ve	−ve	+ve	−ve	+ve	−ve	+ve	−ve	+ve	−ve	+ve	−ve
Slide agglutination	+ve	480	2	286	2	0	0	286	2	0	0	270	1
	−ve	2	656	3	402	0	693	3	402	3	690	2	382

^*a*^ Slide agglutination results are displayed as +ve: positive and −ve: negative.

**TABLE 2:** Sensitivity and specificity of RDTs for detection of *Vibrio cholerae* O1 within suspected cultured colonies as compared to slide agglutination^*a*^

	Compared to slide agglutination	
	Sensitivity (%)	Specificity (%)
Cholkit	99.6	99.7
SD Bioline	99.3	99.3
Crystal VC O1/O139	99.3	99.3
Crystal VC O1	99.6	99.5

^*a*^ The specificity of RDTs for detection of *Vibrio cholerae* O139 within suspected culture colonies was 100% for SD Bioline and 99.6% for Crystal VC O1/O139.

While PCR was performed directly from stool, not from isolated colonies used in culture, PCR results can help contextualize discrepant results between slide agglutination and RDTs. Three samples were positive for *V. cholerae* O139 by Crystal VC O1/O139, with all testing negative by culture/slide agglutination and only one testing positive by PCR (rfbO139 and ctxA positive). There was one instance of a slide agglutination positive sample that was negative by all RDTs (CholKit, SD Bioline, Crystal VC O1/O139, and Crystal VC O1), and *V. cholerae* O1 positive by PCR (rfbO1 positive). Two other samples were positive by slide agglutination, negative by RDT (one negative by CholKit only and one negative by SD Bioline and Crystal VC O1/O139 only), and *V. cholerae* O1 positive by PCR (rfbO1 positive). Four samples were slide-agglutination negative and RDT positive (CholKit, SD Bioline, Crystal VC O1/O139, and Crystal VC O1), with one sample testing negative and the other three testing *V. cholerae* O1 positive by PCR (rfbO1 positive).

## DISCUSSION

Our study found nearly perfect concordance between slide agglutination and RDTs for the identification of O1 serogroup using suspected *V. cholerae* bacterial colonies. These results suggest that RDTs used in this novel way can be an alternative to slide agglutination when antisera and/or well-trained laboratory staff are unavailable.

While not often reported in the formal literature, the lack of culture facilities and maintenance of usable stocks of antiserum in low-resource laboratories is challenging and has led to delayed outbreak confirmations in several countries. The cost of antisera, if purchased commercially, is roughly 4 USD per sample with a shelf life under cold chain of 2 years. In comparison, RDTs typically cost around 2 USD per test (range 2–3 USD with shipping based on pricing within Bangladesh) with a similar shelf life typically at ambient temperatures, making them easier to store and transport and not requiring laboratory infrastructure.

While we aimed to show the concordance of slide agglutination and RDT confirmation using highly trained laboratory technicians, these findings additionally suggest that RDTs have similar, if not superior, practical performance, especially in laboratories where agglutination tests are not routinely performed. Immunochromatographic tests, such as the RDTs used in this study, provide clear color bands to indicate a positive test and a control band to indicate when the test is not working as expected (Fig. S1). In contrast, slide agglutination relies on detecting visible clumping or lower intensities of agglutination, depending on the number of bacteria present, something which can be subjective and may be quite hard to interpret, particularly when technicians have minor vision impairments (Fig. S1).

One limitation of this approach is the inability of these or, to our knowledge, any other commercially available RDTs to identify *V. cholerae* serotype (e.g., Ogawa or Inaba). However, for the purpose of disease surveillance and confirming cholera outbreaks, identifying the serotype of confirmed cases has largely not proven useful, nor is it mentioned in the WHO GTFCC surveillance guidelines. While serotype switching does occur frequently in cholera endemic settings, knowing the serotype will not change control measures or public health decision-making given the high levels of cross-protection expected between serotypes (10). Another limitation of this study is that the same isolate was not used for all four rapid tests and slide agglutination. Given that slide agglutination requires a single colony, it would not be possible to reuse this for the RDTs. However, colonies were all selected from growth on gelatin agar that stemmed from a single bacterial isolate, thus limiting the possibility of differences between isolates.

Our results show that RDTs can be used as an alternative to slide agglutination for the confirmation of *V. cholerae* O1 and may even lead to more consistent and reliable results. As RDTs become more widely available across cholera-affected countries, stockouts of antisera should no longer inhibit confirmation of cholera outbreaks.

## SUPPLEMENTAL MATERIAL

Figure S1 jcm.00433-25-s0001.docx Illustration of a positive (left) and negative (right) sample tested by slide agglutination (top two photos) and RDT (bottom photos). Rapid tests from left to right are CholKit, SD-Bioline, Crystal VC O1/O139, and Crystal VC O1.

ASM does not own the copyrights to Supplemental Material that may be linked to, or accessed through, an article. The authors have granted ASM a non-exclusive, world-wide license to publish the Supplemental Material files. Please contact the corresponding author directly for reuse.
