# Supplementary material for: Rapid tests as a practical alternative to slide agglutination for the confirmation of V. cholerae O1
Source: J Clin Microbiol. 2025 Jul 17;63(8):e00433-25. doi: 10.1128/jcm.00433-25 (PMC12345242; doi:10.1128/jcm.00433-25)
Supplement: Figure S1 — Illustration of a positive (left) and negative (right) sample tested by slide agglutination (top two photos) and RDT (bottom photos). Rapid tests from left to right are CholKit, SD-Bioline, Crystal VC O1/O139, and Crystal VC O1. [file jcm.00433-25-s0001.docx]

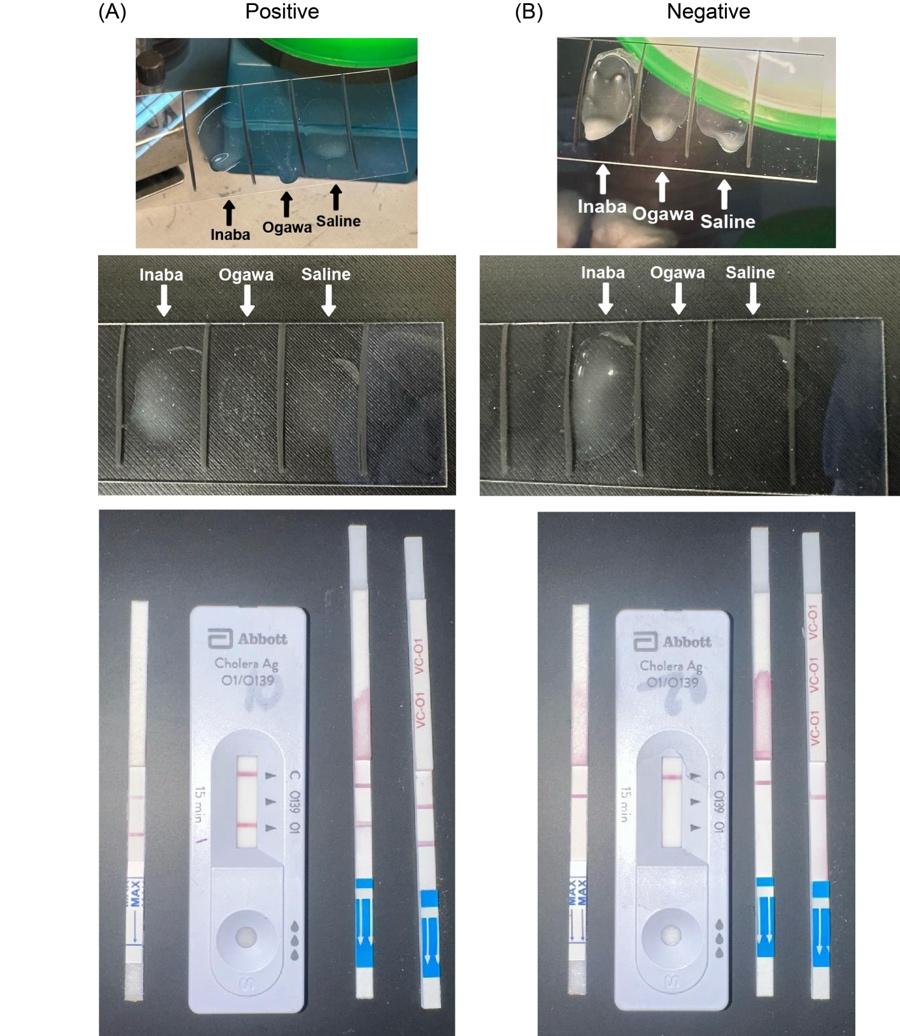


*Figure S 1. Illustration of a positive (left) and negative (right) sample tested by slide agglutination (top two photos) and RDT (bottom photos). Rapid tests from left to right are CholKit, SD-Bioline, Crystal VC O1/O139 and Crystal VC O1.*
